# Aging in Context: The Past, Present, and Future of Research on Older Adults’ Social Ties

**DOI:** 10.1093/geroni/igaa057.1852

**Published:** 2020-12-16

**Authors:** Christina Marini, Stephanie Wilson, Katherine Fiori

## Abstract

This symposium will examine positive and negative aspects of older adults’ relationships and their impacts on health and well-being. We will begin by reviewing the past decade of research on family gerontology. Seidel’s meta-analysis of 995 articles will identify prominent theories and methods, as well as remaining research gaps. The subsequent presentations provide current, cutting-edge research. Marini examines how associations between rumination and sleep unfold within a social context. The findings highlight how spousal support protects older adults’ sleep quality from rumination, whereas support from family and friends is vulnerable to rumination. Using an actor-partner approach, Novak investigates the dynamics of support and control on health among older gay couples. Results reveal the benefits of support and risks of control for partners’ diet quality and depression. Ermer adopts a dyadic perspective to examine links between self-perceptions of aging and inflammation. Results highlight how wives’ inflammation is sensitive to husbands’ aging perceptions, particularly if marital strain is low. Finally, Wilson characterizes age-graded patterns of relationship narratives and their protective effects on emotional well-being. The findings demonstrate how older-adult couples’ narratives are less self- and present-focused, which helps explain protective linkages between age and negative mood. The symposium will conclude with remarks from discussant Katherine Fiori, a GSA Fellow and internationally recognized scholar on older adults’ social networks. She will synthesize the research and put forth her new theory about the importance of peripheral ties in later life to help direct the future of research on older adults within a social context.

